# Genomic determinants of pathogenicity in SARS-CoV-2 and other human coronaviruses

**DOI:** 10.1073/pnas.2008176117

**Published:** 2020-06-10

**Authors:** Ayal B. Gussow, Noam Auslander, Guilhem Faure, Yuri I. Wolf, Feng Zhang, Eugene V. Koonin

**Affiliations:** ^a^National Center for Biotechnology Information, National Library of Medicine, National Institutes of Health, Bethesda, MD 20894;; ^b^Broad Institute of MIT and Harvard, Cambridge, MA 02142; ^c^Howard Hughes Medical Institute, Massachusetts Institute of Technology, Cambridge, MA 02139; ^d^McGovern Institute for Brain Research, Massachusetts Institute of Technology, Cambridge, MA 02139; ^e^Department of Brain and Cognitive Sciences, Massachusetts Institute of Technology, Cambridge, MA 02139;; ^f^Department of Biological Engineering, Massachusetts Institute of Technology, Cambridge, MA 02139

**Keywords:** COVID-19, coronaviruses, pathogenicity, spike protein, nucleocapsid

## Abstract

Severe acute respiratory syndrome coronavirus 2 (SARS-CoV-2) poses an immediate, major threat to public health across the globe. Here we report an in-depth molecular analysis to reconstruct the evolutionary origins of the enhanced pathogenicity of SARS-CoV-2 and other coronaviruses that are severe human pathogens. Using integrated comparative genomics and machine learning techniques, we identify key genomic features that differentiate SARS-CoV-2 and the viruses behind the two previous deadly coronavirus outbreaks, SARS-CoV and Middle East respiratory syndrome coronavirus (MERS-CoV), from less pathogenic coronaviruses. These features include enhancement of the nuclear localization signals in the nucleocapsid protein and distinct inserts in the spike glycoprotein that appear to be associated with high case fatality rate of these coronaviruses as well as the host switch from animals to humans. The identified features could be crucial contributors to coronavirus pathogenicity and possible targets for diagnostics, prognostication, and interventions.

## Significance

The ongoing COVID-19 pandemic is an urgent, global threat. Here we analyze the genome of the virus that causes COVID-19, SARS-CoV-2, along with other members of the coronavirus family. Our analysis identifies crucial genomic features that are unique to SARS-CoV-2 and two other deadly coronaviruses, SARS-CoV and MERS-CoV. These features correlate with the high fatality rate of these coronaviruses as well as their ability to switch hosts from animals to humans. The identified features could represent crucial elements of coronavirus virulence, and allow for detecting animal coronaviruses that have the potential to make the jump to humans in the future.

**Fig. 1.**
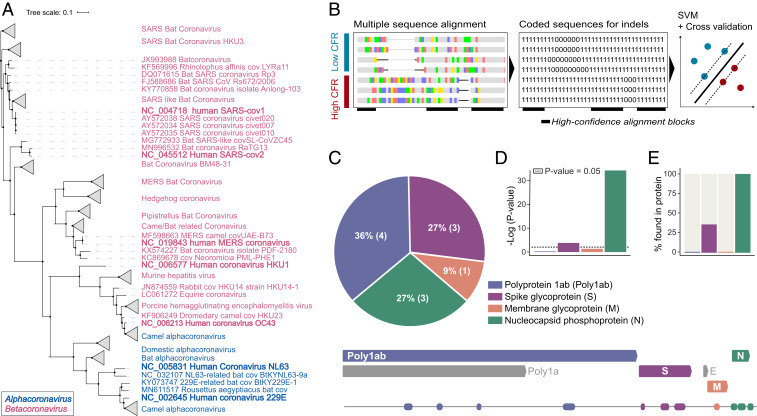
Searching coronavirus genomes for determinants of pathogenicity. (*A*) Phylogenetic tree of coronavirus species, based on the alignment of complete nucleotide sequences of virus genomes. Blue font corresponds to alphacoronaviruses, and magenta font corresponds to betacoronaviruses. (*B*) A schematic illustration of the pipeline applied for detection of genomic regions predictive of high-CFR strains. (*C*) (*Top*) Pie chart showing the percentage of identified genomic determinants in each protein. (*Bottom*) Map of SARS-CoV-2 genome with detected regions. (*D*) Bar plot showing the significance of the distribution of detected regions across each protein. (*E*) Percentage of detected predictive regions in each protein.

The emergence of novel severe acute respiratory syndrome coronavirus 2 (SARS-CoV-2), which causes the respiratory disease coronavirus disease 2019 (COVID-19), triggered a global pandemic that has led to an unprecedented worldwide public health emergency (1). Since it was first reported in December 2019 and as of April 7, 2020, SARS-CoV-2 has infected over a million individuals worldwide, and has led to an estimated 82,000 deaths, with its associated morbidity and mortality cases continuously rising (2). SARS-CoV-2 is the seventh member of the *Coronaviridae* family known to infect humans (3). SARS-CoV and Middle East respiratory syndrome coronavirus (MERS-CoV), two other members of this family, are the causative agents of recent outbreaks, accountable, respectively, for SARS (2002–2003) and MERS (began in 2012) outbreaks (3, 4), and are associated with high case fatality rates (CFR; 9% and 36%, respectively). The novel SARS-CoV-2 can also cause severe disease and is appreciably more infectious than SARS-CoV or MERS-CoV, but with a lower associated CFR (4). By contrast, the other coronaviruses infecting humans, human coronavirus (HCoV)-HKU1, HCoV-NL63, HCoV-OC43, and HCoV-229E, are endemic and cause mild symptoms, accounting for 15 to 29% of common colds (3). The three coronaviruses that can cause severe diseases (hereafter high-CFR coronaviruses) originated in zoonotic transmissions from animal hosts to humans. SARS-CoV and MERS-CoV have bat reservoirs, and were transmitted to humans through intermediate hosts (likely civets and camels, respectively) (4). Similarly, the closest known relative of SARS-CoV-2 is a bat coronavirus (Fig. 1*A*), but the specific route of transmission from bats to humans remains unclear. These repeated, independent zoonotic transmissions and the high associated pathogenicity call for an in-depth investigation of the genomic features that contribute to coronaviruses pathogenicity and transmission, to better understand the molecular mechanisms of the high-CFR coronaviruses pathogenicity, and thus to be better prepared for any future coronavirus outbreaks, and potentially contribute to the development of interventions.

## Results and Discussion

### Detection of Diagnostic Features of High-CFR Coronaviruses.

In this work, we developed an approach combining advanced machine learning methods with well-established genome comparison techniques, to identify the potential genomic determinants of pathogenicity of the high-CFR coronavirus strains (Fig. 1*B*). Coronaviruses have positive-sense RNA genomes consisting of six conserved proteins, along with additional strain-specific accessory proteins (5). The conserved proteins are the polyproteins pp1a and pp1ab that encompass multiple protein domains involved in various aspects of coronavirus genome replication, spike glycoprotein (S), envelope (E), membrane glycoprotein (M), and nucleocapsid phosphoprotein (N) (Fig. 1*C*). To detect potential genomic determinants of pathogenicity, we first aligned the complete genomes of all human coronaviruses (*n* = 944 genomes; Deposited data file 1) (6), and coded the alignment to specifically distinguish deletions and insertions (Fig. 1*B*; see *Methods* for details).

We hypothesized that the high-CFR coronavirus strains are more pathogenic due to shared genomic determinants that are absent in the low-CFR strains. To test this hypothesis, we applied dimensionality reduction to the coded, aligned coronaviruses genomes (see *Methods* for details), and visualized the results to evaluate whether this representation of the genomes resulted in clustering by the CFR trait (*SI Appendix*, Fig. S1). Phylogenetically, the human coronaviruses span two genera, *Alphacoronavirus* and *Betacoronavirus*, with the low-CFR strains spanning both genera and the high-CFR strains belonging solely to *Betacoronavirus* (5) (Fig. 1*A*). Despite this phylogenetic segregation, the dimensionality reduction analyses cluster the SARS clade separately, and all other coronaviruses as one cluster. Nevertheless, the MERS-CoV strains occupied intermediate positions between the clusters (albeit closer to the low-CFR coronaviruses), indicating that the CFR trait might impact the clustering of coronaviruses by deletions and insertions. We reasoned that specific regions within the alignment should exist that would reflect a clustering where the intermediate MERS-CoV strains group together with the other high-CFR strains, and that such regions would classify the viruses based on CFR. We sought to identify such genomic regions that are conserved among the members of each group (high-CFR or low-CFR) but not conserved when comparing between the groups. Because the high-CFR strains share a common ancestor (of both the SARS and the MERS clades), any detected regions that classify coronaviruses based on CFR either might have emerged in the high-CFR common ancestor or might have evolved independently in different high-CFR strains. These distinct evolutionary scenarios would have different implications for the evolution of the enhanced pathogenicity of the high-CFR strains. To identify such features in a statistically robust fashion, while considering all available genomes, we used machine learning (see *Methods* for details). Specifically, we trained multiple support vector machines (SVMs) across a sliding window to detect regions that confer clean separation between high- and low-CFR virus genomes. We evaluated the performance of each SVM via cross-validation and filtered for genomic regions that significantly distinguish the high- and low-CFR genomes. This approach enables automatic detection of regions with subtle differences between high- and low-CFR coronaviruses genomes that are not easily distinguishable with straightforward alignment analysis techniques (7).

In total, our method identified 11 regions of nucleotide alignments that were reliably predictive of the high CFR of coronaviruses (Fig. 1*C* and *SI Appendix*, Table S1). These regions occurred in four proteins, namely, pp1ab (peptides nsp3, nsp4, and nsp14), spike glycoprotein, membrane glycoprotein, and nucleocapsid. Two proteins were significantly enriched with these predictive regions: the nucleocapsid protein and the spike glycoprotein (*P* values: 4e-16 and 0.036, respectively; Fig. 1*D*). Only four of the diagnostic regions detected in the nucleotide alignment corresponded to observable insertions and deletions in the protein alignments as well, with three located in the nucleocapsid protein and one in the spike protein (Fig. 1*E*). We therefore focus our analyses on these two proteins.

### Enhancement of Nuclear Localization Signals in the Nucleocapsid Protein.

**Fig. 2.**
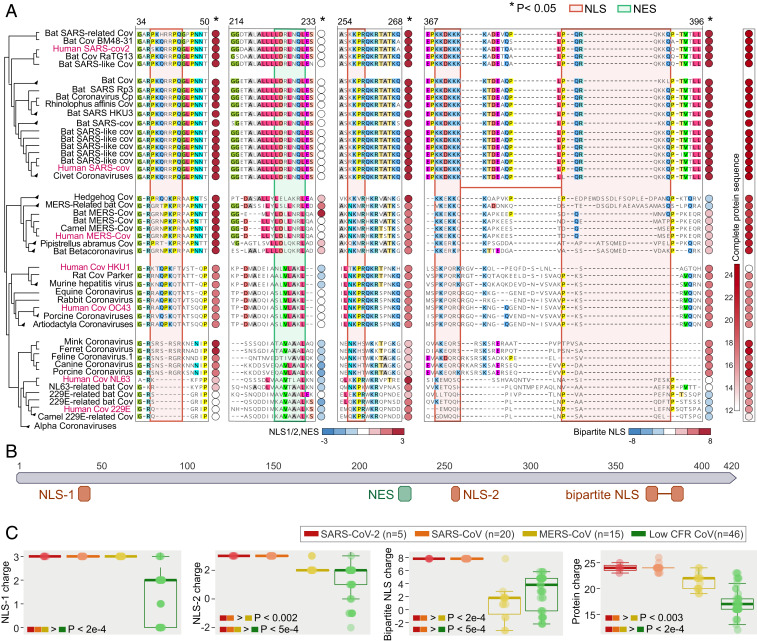
Putative determinants of coronavirus pathogenicity in the nucleocapsid and the spike protein. (*A*) (*Left*) Phylogenetic tree and protein alignment of the nucleocapsid protein across coronavirus species with the unrooted tree built based on the nucleocapsid amino acid sequences. NLS and NES are outlined in orange and green, respectively. The circle next to each signal sequence denotes peptide charge, with red denoting higher charge and blue denoting a lower charge. (*Right*) The overall charge of each full protein sequence. (*B*) Map of SARS-CoV-2 nucleocapsid protein with relevant NLS (orange) and NES (green) motifs marked. (*C*) Boxplots displaying (*Left*, *Center Left*, and *Center Right*) the charge of the three NLS motifs and (*Right*) that of the complete nucleocapsid protein for SARS-CoV-2, SARS-CoV, MERS-CoV, and low-CFR strains. The one-sided rank sum *P* values are shown when significant between any two groups, supporting a gradual increase of the charge.

Exploring the regions identified within the nucleocapsid that predict the high CFR of coronaviruses, we found that these deletions and insertions result in substantial enhancement of motifs that determine nuclear localization (8), specifically, in high-CFR coronaviruses (*SI Appendix*, Fig. S2*A*). The deletions, insertions, and substitutions in the N proteins of the high-CFR coronaviruses map to two monopartite nuclear localization signals (NLSs), one bipartite NLS and a nuclear export signal (NES) (Fig. 2 *A* and *B*). In the course of the evolution of coronaviruses, these nuclear localization and export signals grow markedly stronger in the clades that include the high-CFR viruses and their relatives from animals (primarily, bats), as demonstrated by the increasing positive charge of the amino acids comprising the NLS, a known marker of NLS strength (9) (Fig. 2*A*). In the clade that includes SARS-CoV and SARS-CoV-2, the accumulation of positive charges was observed in the monopartite NLS, the bipartite NLS, and the NES, whereas, in the clade including MERS-CoV, positive charges accumulated primarily in the first of the two monopartite NLSs (Fig. 2*A* and *SI Appendix*, Table S2). In all cases, the enhancement of these signals is a gradual, significant trend that accompanied coronavirus evolution concomitantly with the emergence of more pathogenic strains (empirical *P* value < 0.001; Fig. 2 *A* and *C*). The charge of the complete nucleocapsid protein gradually evolves toward greater positive values due, specifically, to the formation of the NLS, as demonstrated by sequence permutation analysis (Fig. 2*A*; see *Methods* for details), which implies a key role for these motifs in the function of the nucleocapsid, including potential contribution to virus pathogenicity. The N protein is multifunctional, contributing to viral transcription efficiency and pathogenesis, and interacts with both the genomic RNA and the M protein (10). Therefore, the increasing charge could affect any or all of these functions. This potential pleiotropy notwithstanding, the accumulation of positive charges directly strengthens the NLS (9), which correlates with the growing CFR of coronaviruses (Fig. 2*C*). The implication of these observations thus is that the localization pattern of the nucleocapsid proteins of high-CFR strains differs from that of the low-CFR strains and might contribute to the increased pathogenicity of the high-CFR strains. Localization of the nucleocapsid protein to the nuclei, and, specifically, to the nucleoli, has been previously reported in coronaviruses (11) and has been associated with increased pathogenicity in a porcine coronavirus model (10, 12, 13). The presence of both NLS and NES raises an uncertainty as to the precise effect of these motifs on the nucleocapsid protein localization, and the reports are indeed somewhat contradictory (8, 10, 14). Nevertheless, the striking extent of the changes in the NLS of the high-CFR strains (Fig. 2*C*) suggests that localization of the nucleocapsid protein could be an important determinant of coronavirus pathogenicity.

### A Unique Insertion Upstream of the Heptad Repeat Region of the Spike Glycoprotein in High-CFR Coronaviruses.

**Fig. 3.**
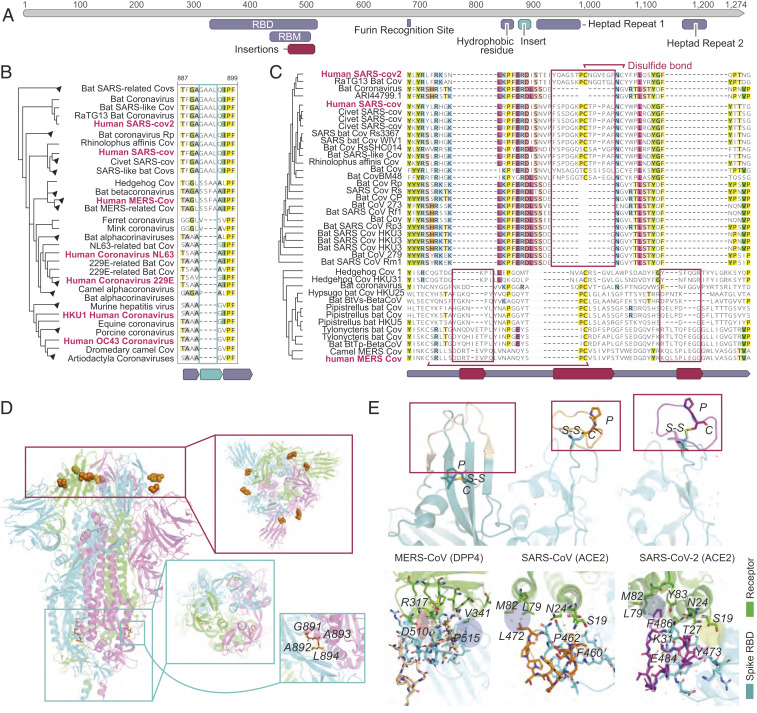
The signature inserts in the spike glycoproteins of the high-CFR coronaviruses. (*A*) Map of SARS-CoV-2 spike protein with relevant protein regions and the features detected by the present analysis with the unrooted tree built based on the spike amino acid sequences. The relevant regions include the RBD, the RBM, the furin recognition site, a hydrophobic residue preceding the first heptad repeat, and both heptad repeats. The two features detected by this analysis are the insertions in the RBM found in pathogenic strains before the zoonotic transmission to human, and the insertion in the high-CFR strains preceding the heptad repeat. (*B*) Phylogenetic tree and protein alignment of the spike protein insertion preceding the first heptad repeat with the unrooted tree built based on the spike amino acid sequences. (*C*) Phylogenetic tree and protein alignment of the spike protein zoonotic insertions in the RBM of high-CFR coronaviruses (disulfide bonds are shown for human strains). (*D*) Structure of the SARS-CoV-2 spike glycoprotein trimer with the inserts mapped to the heptad repeat-containing domain and the receptor-binding domain. *Top Inset* (red rectangle) shows the locations of the inserts in the RBM, designated as in *A*, that are located within segments bordered by orange spheres (unresolved in the structure). *Middle Inset* (blue rectangle) shows the GAAL insert upstream of the first heptad-repeat region. *Bottom Inset* (blue rectangle) shows a close view of the GAAL insert (in orange). (*E*) (*Top*) Structures of the receptor-binding motifs of SARS-CoV, SARS-CoV-2 and MERS-CoV. The inserts are highlighted in wheat for MERS-CoV, orange for SARS-CoV, and purple for SARS-CoV-2, and the PC doublets and disulfide bonds are shown. (*Bottom*) Interactions between the inserts in the RBM of the spike glycoproteins of SARS-CoV, SARS-CoV-2, and MERS-CoV, and the corresponding human receptors. Residues shown with stick models are within a 5-Å distance from the interacting residues in the inserts. The salt bridge is highlighted in red with a thick red border (in MERS-CoV), charge interaction is highlighted in red with a thin blue border (SARS-CoV-2), and H-bond network is highlighted in yellow (Y473, T27, S19).

We next investigated the diagnostic feature identified within the spike glycoprotein. The SARS-CoV-2 spike protein binds ACE2, the host cell receptor of SARS-CoV-2 (15), with a 10- to 20-fold greater affinity compared to SARS-CoV, and contains a polybasic furin cleavage site resulting from a unique insert to SARS-CoV-2 that could enhance infectivity (4). The spike protein consists of multiple domains (15) (Fig. 3*A*), including two heptad repeat regions that are crucial to infection (16). During membrane fusion, the heptad repeats fold into a six-helical bundle that forms the stable fusion core which facilitates the insertion of the hydrophobic fusion peptide into the host membrane and brings the viral and host membranes into proximity as required for fusion (17–19). The spike protein fusion peptide is located upstream of the first heptad repeat (20, 21) (HR1), with a long connecting region between the fusion peptide and HR1 that adopts an α-helical structure. Our analysis revealed a four-amino acid insertion in the connecting region in all high-CFR viruses but not in any of the low-CFR ones, with the MERS and SARS clades apparently acquiring this insertion independently, as supported by the unrelated insert sequences (Fig. 3*B* and *SI Appendix*, Fig. S2*B*). The insertion increases the length and flexibility of the connecting region as confirmed by the examination of the spike glycoprotein structure of SARS-CoV (Fig. 3*D*), and, therefore, is likely to affect the fusion process, although the specific contribution of this insert to pathogenicity remains to be studied experimentally.

### Unique Inserts Associated with Zoonotic Jumps to Humans in the Spike Glycoprotein.

Finally, we sought to identify genomic features that might be associated with the repeated jumping of coronaviruses across the species barriers to humans, specifically, in the case of the high-CFR strains. To this end, we aligned the genomes of all coronaviruses from different hosts (Deposited data file 2) and selected, for each human-infecting strain of the high-CFR coronaviruses, the closest nonhuman infecting relatives (see *Methods* for details). Within each such set of human high-CFR coronaviruses and their animal ancestors, we searched for genomic insertions or deletions that occurred in the most proximal strains before the zoonotic jump to humans. This analysis identified independent insertions in each of the three groups of viruses, all of which were located within the spike glycoprotein, specifically, in the receptor-binding domain (RBD), within the subdomain that binds ACE2 (15, 22) (the receptor-binding motif [RBM]) in the cases of SARS-CoV and SARS-CoV-2, and DPP4 in the case of MERS-CoV (23) (Fig. 3 *C* and *D*).

The insertions occur in slightly different locations within the RBM structure, with a single insert in the SARS clade and two distinct inserts in the MERS clade, and show no sequence similarity between the three high-CFR groups, suggesting independent evolutionary events. The two insertions in the MERS clade correspond to two loops connecting the distal β-strand of an extended β-sheet structure, whereas the insert in the SARS clade corresponds to a single long loop embedded within a short, unstable β-sheet. Despite the lack of an overall similarity, in each case, the inserted segments contain a proline−cysteine (PC) amino acid doublet (Fig. 3 *C* and *E* and *SI Appendix*, Fig. S2). In both high-CFR coronavirus clades, the cysteines in the insert form disulfide bonds with other cysteines in the RBM, although the locations of the partner cysteines are different, that is, proximal, within the loop containing the insert in SARS, and distal, within the RBM β-sheet in MERS (Fig. 3*E* and *SI Appendix*, Fig. S3). These different locations of the disulfide bridges result in distinct RBM conformations (Fig. 3*E*) corresponding to the different receptor specificities in human cells. In both SARS and MERS, the insert directly interacts with the respective receptors but the specifics of the interactions differ, with a salt bridge (D510 with R317) and a hydrophobic interaction (P515 with V341) in the case of MERS-CoV, and a hydrophobic interaction patch in SARS-CoV and SARS-CoV-2 each, involving L472 and F486, respectively. The flexibility added to the RBM by these inserts could allow the spike to be more malleable in binding to a receptor, allowing for zoonotic transmission. Furthermore, the SARS inserts that are located within a short, unstable β-sheet provide more flexibility than the MERS inserts which are within longer, more rigid β-sheets, potentially contributing to MERS-CoV never fully adapting to human-to-human transmission (4). The phenylalanine in SARS-CoV-2 provides a larger hydrophobic surface to interact with three residues of ACE2 (M82, L79, and Y83), whereas, in SARS-CoV, the leucine interacts with two residues (M82 and L79). The SARS-CoV-2 insert also engages in an extra charge interaction with ACE2 (E484 with K31) and multiple hydrogen bonds (Fig. 3*D*). The larger hydrophobic surface and the additional interactions could, in part, underlie the higher binding affinity of SARS-CoV-2 to ACE2 compared to SARS-CoV^13^. Thus, the independent insertions in the RBM of the spike protein are highly likely to contribute to or even enable the zoonotic transmission of the high-CFR coronavirus strains to humans and might also contribute to their high CFR.

## Conclusions

SARS-CoV-2 has led to the most devastating pandemic since the 1918 Spanish flu, prompting an urgent need to elucidate the evolutionary history and genomic features that led to the increased pathogenicity and rampant spread of this virus as well as those coronaviruses that caused previous deadly outbreaks. A better understanding of viral pathogenicity and zoonotic transmission is crucial for prediction and prevention of future outbreaks. Here, using an integrated approach that included machine-learning and comparative genomics, we identified three previously undetected likely determinants of pathogenicity and zoonotic transmission. The enhancement of the NLS in the high-CFR coronaviruses nucleocapsids implies an important role of the subcellular localization of the nucleocapsid protein in coronavirus pathogenicity. Strikingly, insertions in the spike protein appear to have been acquired independently by the SARS and MERS clades of the high-CFR coronaviruses, in both the domain involved in virus−cell fusion and the domain mediating receptor recognition. The gradual enhancement of the NLS in the nucleocapsids and the different insertions in the spike protein of the high-CFR coronaviruses imply that these changes do not reflect a single event that occurred in the common ancestor, but rather a convergent trend in the evolution of the high-CFR viruses. These insertions, most likely, enhance the pathogenicity of the high-CFR viruses and contribute to their ability to zoonotically transmit to humans. All of these features are shared by the high-CFR coronaviruses and their animal (in particular, bat) infecting relatives in the same clade, which demonstrates that the emergence of SARS-CoV-2 is a natural part of the ongoing coronavirus evolution and is compatible with the possibility of future zoonotic transmission of additional highly pathogenic strains to humans. The predictions made through this analysis unveil potential critical features in the mechanism of SARS-CoV-2 virulence and its evolutionary history, are amenable to straightforward experimental validation, and could serve as predictors of strains pathogenic to humans.

## Methods

### Data.

The complete nucleotide sequences of 3,001 coronavirus genomes were obtained from National Center for Biotechnology Information (NCBI) (Deposited data file 2). Of these, 944 genomes belong to viruses that infect humans, including both viruses with low CFR, HCoV-NL63, HCoV-229E, HCoV-OC43, and HCoV-HKU1, and those with high CFR, namely, MERS-CoV, SARS-CoV, and SARS-CoV-2. The protein sequences that are encoded in the genomes of all human coronaviruses and closely related viruses from animals were obtained from NCBI, including the two polyproteins (1ab and 1a), spike glycoprotein, envelope, membrane glycoprotein, and nucleocapsid phosphoprotein.

### Alignment Representation.

We aimed to represent the sequences in a way that would enable evaluation across different strains and viruses. To this end, the 944 human coronavirus genomes were aligned using MAFFT (a multiple sequence alignment program for unix-like operating systems) (24) v7.407. The aligned sequences were then recoded such that each nucleotide was coded as “1” and each gap was coded as “0.” This representation allows identification of insertions and deletions only, which was the focus of the analysis and methodology employed here. Incorporation of substitution mutations would require a more complex representation of the data, which is outside of the scope of this study.

### Dimensionality Reduction.

To examine how the coded alignment (representing insertions and deletions) clusters the different coronaviruses, two dimensionality reduction approaches were applied: 1) principal component analysis (PCA), using the Python library scikit-learn (25) decomposition PCA function with two dimensions, and 2) t-distributed stochastic neighbor embedding (tSNE), using the Python library scikit-learn manifold tSNE function with two dimensions and perplexity set to 50. We then visualized the data in the reduced dimensions obtained from the two approaches (*SI Appendix*, Fig. S1). We performed *k*-means clustering on the reduced-dimensionality data (using the Python library scikit-learn cluster KMeans function with *k* = 2), and found that the SARS clade segregated to a separate cluster, whereas MERS-CoV came across as an intermediate between SARS and the low-CFR strains, closer to most of the low-CFR strains.

### Identification of Genomic Determinants of High-CFR Coronaviruses.

Given the finding that the coded alignment clustering of the data were strongly influenced by the CFR trait, we next searched for specific regions within this coded alignment that contributed the most to the separation between high- and low-CFR viruses. Specifically, we searched for regions that could be used to accurately differentiate between high- and low-CFR strains. To this end, comparative genomics was combined with machine learning techniques. We used the coded alignment and applied the following steps:

1) High-confidence alignment blocks were identified within the multiple sequence alignment (MSA), which were defined as regions longer than 15 nt, containing less than 40% gaps in each position. The following steps were applied only within those high-confidence alignment blocks, because these regions (spanning 53% of the total alignment) are most likely to contain relevant differences within conserved genomic regions.
2) We then trained SVMs [using the Python library scikit-learn (25) with a linear kernel function] on all 5-nt sliding windows in the identified high-confidence alignment regions, using a cross-validation technique. To account for the different sample sizes available for each virus (*SI Appendix*, Table S3), cross-validation with seven folds was applied. In each fold, all of the samples of one of the seven coronaviruses were left out, an SVM was trained on all samples of the remaining six viruses, and it was then tested on the left-out samples.
3) For each of these sliding windows, the seven accuracy values obtained by the seven folds were evaluated, and windows where all accuracy values were greater than 80% were considered further. This high threshold was set to overcome the unbalanced phylogeny of the strains and to distinguish alignment regions that were consistent within high-CFR strains and low-CFR strains, but differed between the two.
4) The regions that could classify the high- vs. low-CFR viruses with this high level of accuracy were each considered and examined, to define the precise borders of each region. From the 11 regions identified, 4 were in polyprotein 1ab, 3 were in the spike glycoprotein, 1 was in the membrane glycoprotein, and 3 were in the nucleocapsid phosphoprotein.

### Significance Evaluation.

To evaluate the significance of these findings, we computed a hypergeometric enrichment *P* value, using the sizes of the identified regions and the lengths of the coding regions of each protein within the MSA. We found that the nucleocapsid phosphoprotein was most enriched with genomic differences that predict CFR (*P* value = 4e-16), followed by the spike glycoprotein (*P* value = 0.036), and that the polyprotein 1ab and membrane glycoprotein were not significantly enriched with such differences. We further examined the effects of this set of genomic differences on the resulting protein sequences, and found that only 4 of the 11 differences identified were reflected in the protein alignment, of which 3 occurred in the nucleocapsid phosphoprotein and 1 in the spike glycoprotein.

### Nonhuman Proximal Coronavirus Strains.

To compile a list of human and proximal nonhuman coronavirus strains, we first constructed an MSA of all 3,001 collected strains using MAFFT v7.407. From that alignment, we built a phylogenetic tree using FastTree (26) 2.1.10 with the “-nt” parameter, and extracted the distances between leaves of each strain from each of the reference genomes of the seven human coronaviruses (Deposited data file 3). We then extracted the proximal strains of each human coronavirus, which were within a distance of less than 1.0 substitution per site to one of the human coronaviruses. To obtain a unique set of strains, we removed highly similar strains by randomly sampling one strain from each group of strains with more than 98% pairwise sequence identity (the resulting strains are provided in Deposited data file 4).

### Amino Acid Charge Calculations.

To evaluate the strength of the identified NLS and NES motifs within the nucleocapsid phosphoprotein, we calculated the amino acid cumulative charge within the alignment region of each motif, and of the complete protein, for each of the selected human and proximal nonhuman coronavirus strains (Deposited data file 4). The charge of each region was evaluated by the number of positively charged amino acids (lysine and arginine) minus the number of negatively charged amino acids (aspartic acid and glutamic acid) in the region. To evaluate the significance of the association between CFR and the charge of specific motifs within the nucleocapsid phosphoprotein, we first calculated the rank sum *P* value comparing the charges of regions in high-CFR versus low-CFR strains. Then, we applied a permutation test, by counting the fraction of similar or more significant charge differential values between high-CFR and low-CFR viruses among 1,000 randomly selected motifs of similar length from the alignment of the nucleocapsid phosphoprotein.

### Genomic Determinants of the Interspecies Jump.

To identify genomic determinants that discriminate high-CFR viruses that made the zoonotic transmission to humans, we used the nucleotide MSA of MERS-CoV, SARS-CoV, and SARS-CoV-2 and the selected proximal nonhuman coronaviruses of each of these.

We searched regions that maximize the following function:

$$f\left(S,V\right)=\underset{d_{k}:k\;\mathrm{in}\;V}{min}\prod_{j:d_{j}>d_{k}}I_{S_{j}\neq S_{h}}$$

where

$$I_{S_{j}\neq S_{h}}=\left\{ \begin{array}{c} 1\;if\;S_{j}\neq S_{h}\\ 0\;else \end{array}\right..$$

S is a position within the encoded MSA (“1” for a nucleotide and “0” for a gap), and V is the set of strains selected for either MERS-CoV, SARS-CoV, or SARS-CoV-2; $d_{k}$is the distance of nonhuman strain k from the human strain of group V, $S_{j}$ is position S of nonhuman strain j, and $S_{h}$ is position S of the human strain in group V.

Thus, this function aims to find, for each position, within each of the three groups of strains, the nonhuman strain k with the minimal distance from the human strain, such that all nonhuman strains that are more distant are more different from the human strain in that position (i.e., a genomic change that occurred as close as possible to the human strain). We searched for regions in which over 50% of the strains in the alignment differed from the human strain, and for which the differing strains were explicitly the most distant from human. We identified only one such location, across all three high-CFR virus groups.

### Structural Analysis of the Spike Glycoproteins−Receptor Complexes.

Crystal structures of the RBDs of the spike glycoproteins of SARS-CoV (Protein Data Bank [PDB] ID code 2ajf) (27), SARS-CoV-2 (PDB ID code 6m0j) (28) and MERS-CoV (PDB ID code 4l72) (23) complexed with their respective receptors, and the full cryoelectron microscopy structure of the SARS-CoV-2 spike glycoprotein (PDB ID code 6vxx) (29) were downloaded from the PDB (30). Structural analyses including residues interactions and structural alignments were performed using the PyMOL computational framework (31).

### Data Availability Statement.

All data used in this work are publicly available. The alignments files used throughout this work are deposited at https://zenodo.org/record/3832484 (6).

## Supplementary Material

Supplementary File

## References

[1] World Health Organization, “Statement on the second meeting of the International Health Regulations (2005) Emergency Committee regarding the outbreak of novel coronavirus (2019-nCoV)” (World Health Organization, 2020).

[2] World Health Organization, Coronavirus disease (COVID-19) Pandemic. https://www.who.int/emergencies/diseases/novel-coronavirus-2019. Accessed 1 April 2020.

[3] Su S.., Epidemiology, genetic recombination, and pathogenesis of coronaviruses. Trends Microbiol. 24, 490–502 (2016). 27012512 10.1016/j.tim.2016.03.003 PMC7125511

[4] Zhang Y.-Z., Holmes E. C., A genomic perspective on the origin and emergence of SARS-CoV-2. Cell 181, 223–227 (2020). 32220310 10.1016/j.cell.2020.03.035 PMC7194821

[5] Cui J., Li F., Shi Z.-L., Origin and evolution of pathogenic coronaviruses. Nat. Rev. Microbiol. 17, 181–192 (2019). 30531947 10.1038/s41579-018-0118-9 PMC7097006

[6] Gussow A. B., Genomic determinants of pathogenicity in SARS-CoV-2 and other human coronaviruses. Zenodo. https://zenodo.org/record/3832484. Deposited 18 May 2020. 10.1073/pnas.2008176117 PMC7334499 32522874

[7] Auslander N., Wolf Y. I., Shabalina S. A., Koonin E. V., A unique insert in the genomes of high-risk human papillomaviruses with a predicted dual role in conferring oncogenic risk. F1000 Res. 8, 1000 (2019). 10.12688/f1000research.19590.1 PMC6685453 31448109

[8] You J.., Subcellular localization of the severe acute respiratory syndrome coronavirus nucleocapsid protein. J. Gen. Virol. 86, 3303–3310 (2005). 16298975 10.1099/vir.0.81076-0

[9] Cokol M., Nair R., Rost B., Finding nuclear localization signals. EMBO Rep. 1, 411–415 (2000). 11258480 10.1093/embo-reports/kvd092 PMC1083765

[10] McBride R., van Zyl M., Fielding B. C., The coronavirus nucleocapsid is a multifunctional protein. Viruses 6, 2991–3018 (2014). 25105276 10.3390/v6082991 PMC4147684

[11] Wurm T.., Localization to the nucleolus is a common feature of coronavirus nucleoproteins, and the protein may disrupt host cell division. J. Virol. 75, 9345–9356 (2001). 11533198 10.1128/JVI.75.19.9345-9356.2001 PMC114503

[12] Chen H., Wurm T., Britton P., Brooks G., Hiscox J. A., Interaction of the coronavirus nucleoprotein with nucleolar antigens and the host cell. J. Virol. 76, 5233–5250 (2002). 11967337 10.1128/JVI.76.10.5233-5250.2002 PMC136173

[13] Pei Y.., Functional mapping of the porcine reproductive and respiratory syndrome virus capsid protein nuclear localization signal and its pathogenic association. Virus Res. 135, 107–114 (2008). 18403041 10.1016/j.virusres.2008.02.012

[14] Rowland R. R. R.., Intracellular localization of the severe acute respiratory syndrome coronavirus nucleocapsid protein: Absence of nucleolar accumulation during infection and after expression as a recombinant protein in vero cells. J. Virol. 79, 11507–11512 (2005). 16103202 10.1128/JVI.79.17.11507-11512.2005 PMC1193611

[15] Wrapp D.., Cryo-EM structure of the 2019-nCoV spike in the prefusion conformation. Science 367, 1260–1263 (2020). 32075877 10.1126/science.abb2507 PMC7164637

[16] Bosch B. J.., Severe acute respiratory syndrome coronavirus (SARS-CoV) infection inhibition using spike protein heptad repeat-derived peptides. Proc. Natl. Acad. Sci. U.S.A. 101, 8455–8460 (2004). 15150417 10.1073/pnas.0400576101 PMC420415

[17] Chan W.-E., Chuang C.-K., Yeh S.-H., Chang M.-S., Chen S. S.-L., Functional characterization of heptad repeat 1 and 2 mutants of the spike protein of severe acute respiratory syndrome coronavirus. J. Virol. 80, 3225–3237 (2006). 16537590 10.1128/JVI.80.7.3225-3237.2006 PMC1440416

[18] Du L.., The spike protein of SARS-CoV—A target for vaccine and therapeutic development. Nat. Rev. Microbiol. 7, 226–236 (2009). 19198616 10.1038/nrmicro2090 PMC2750777

[19] Lu L.., Structure-based discovery of Middle East respiratory syndrome coronavirus fusion inhibitor. Nat. Commun. 5, 3067 (2014). 24473083 10.1038/ncomms4067 PMC7091805

[20] Lai A. L., Millet J. K., Daniel S., Freed J. H., Whittaker G. R., The SARS-CoV fusion peptide forms an extended bipartite fusion platform that perturbs membrane order in a calcium-dependent manner. J. Mol. Biol. 429, 3875–3892 (2017). 29056462 10.1016/j.jmb.2017.10.017 PMC5705393

[21] Millet J. K., Whittaker G. R., Physiological and molecular triggers for SARS-CoV membrane fusion and entry into host cells. Virology 517, 3–8 (2018). 29275820 10.1016/j.virol.2017.12.015 PMC7112017

[22] Wan Y., Shang J., Graham R., Baric R. S., Li F., Receptor recognition by the novel coronavirus from Wuhan: An analysis based on decade-long structural studies of SARS coronavirus. J. Virol. 94, e00127-20 (2020). 31996437 10.1128/JVI.00127-20 PMC7081895

[23] Wang N.., Structure of MERS-CoV spike receptor-binding domain complexed with human receptor DPP4. Cell Res. 23, 986–993 (2013). 23835475 10.1038/cr.2013.92 PMC3731569

[24] Katoh K., Standley D. M., MAFFT multiple sequence alignment software version 7: Improvements in performance and usability. Mol. Biol. Evol. 30, 772–780 (2013). 23329690 10.1093/molbev/mst010 PMC3603318

[25] Pedregosa F.., Scikit-learn: Machine learning in Python. J. Mach. Learn. Res. 12, 2825–2830 (2011).

[26] Price M. N., Dehal P. S., Arkin A. P., FastTree 2—Approximately maximum-likelihood trees for large alignments. PLoS One 5, e9490 (2010). 20224823 10.1371/journal.pone.0009490 PMC2835736

[27] Li F., Li W., Farzan M., Harrison S. C., Structure of SARS coronavirus spike receptor-binding domain complexed with receptor. Science 309, 1864–1868 (2005). 16166518 10.1126/science.1116480

[28] Lan J.., Structure of the SARS-CoV-2 spike receptor-binding domain bound to the ACE2 receptor. Nature 581, 215–220 (2020). 32225176 10.1038/s41586-020-2180-5

[29] Walls A. C.., Structure, function, and antigenicity of the SARS-CoV-2 spike glycoprotein. Cell 181, 281–292.e6 (2020). 32155444 10.1016/j.cell.2020.02.058 PMC7102599

[30] Berman H. M.., The Protein Data Bank. Nucleic Acids Res. 28, 235–242 (2000). 10592235 10.1093/nar/28.1.235 PMC102472

[31] Schrödinger LLC, The PyMOL Molecular Graphics System (Version 2.0, Schrödinger LLC).

