# Supplementary material for: Genomic determinants of pathogenicity in SARS-CoV-2 and other human coronaviruses
Source: Proc Natl Acad Sci U S A. 2020 Jun 10;117(26):15193–9. doi: 10.1073/pnas.2008176117 (PMC7334499; doi:10.1073/pnas.2008176117)
Supplement: Supplementary File [file pnas.2008176117.sapp.pdf]

Genomic determinants of pathogenicity in SARS-CoV-2 and other human coronaviruses

Ayal B. Gussow[1,*], Noam Auslander[1,*,#], Guilhem Faure[2], Yuri I. Wolf[1], Feng Zhang[2,3,4,5], Eugene V. Koonin[1,#]

[1]National Center for Biotechnology Information, National Library of Medicine, National Institutes of Health, Bethesda, MD 20894, USA
[2]Broad Institute of MIT and Harvard, Cambridge, MA, USA
[3]McGovern Institute for Brain Research, Massachusetts Institute of Technology, Cambridge, MA 02139, USA
[4]Department of Brain and Cognitive Sciences, Massachusetts Institute of Technology, Cambridge, MA 02139, USA
[5]Department of Biological Engineering, Massachusetts Institute of Technology, Cambridge, MA 02139, USA

* These authors contributed equally
[#]For correspondence: koonin@ncbi.nlm.nih.gov; noam.auslander@nih.gov

Supporting Information

Supporting Tables

Alignment location				
**start**	**end**	**gene**	Loc [SARS-Cov-2]	**type**
7281	7291	pp1ab (nsp3)	3728	indel
10446	10451	pp1ab (nsp3)	6437	indel
14249	14249	pp1ab (nsp4)	9750	indel
25041	25108	pp1ab (nsp14)	19021	indel
29498	29498	S (spike glycoprotein)	22358	indel
32029	32040	S (spike glycoprotein)	24228	indel
32906	32927	S (spike glycoprotein)	25001	indel
36459	36462	M (membrane glycoprotein)	27001	indel
38798	38808	N (nucleocapsid)	29114	indel
38926	38941	N (nucleocapsid)	29235	indel
39356	39360	N (nucleocapsid)	29534	indel

Supporting Table 1. The regions detected from the nucleotide genome alignment. The first two columns indicate the start and end positions of the detected region within the nucleotide genome alignment. Coordinates are inclusive. The third column indicates the gene that the region occurs in, the fourth column is the coordinate of the region in the SARS-CoV-2 reference, and the final column is the type of region detected.

	Motif1 (NLS1)	Motif2 (NES)	Motif3 (NLS2)	Motif4 (bipartite NLS)	Protein charge
YP_009724397	3	0	3	8	24
QHR63308	3	0	3	8	24
AVP78038	3	0	3	8	25
ASO66816	3	0	3	8	24
ABD75315	3	0	3	8	23
ABG47067	3	0	3	8	24
Q3I5I7	3	0	3	8	24
AGC74175	3	0	3	8	24
AHX37566	3	0	3	8	23
AAZ41337	3	0	3	8	24
Q3LZX4	3	0	3	8	24
ADE34730	3	0	3	8	23

AGC74169	3	0	3	8	24
AAZ67039	3	0	3	8	24
Q0Q468	3	0	3	8	26
ACU31039	3	0	3	8	25
ATO98190	3	0	3	8	24
AGZ48815	3	0	3	8	24
ARI44802	3	0	3	8	24
AGZ48841	3	0	3	8	24
NP_828858	3	0	3	8	24
AAU04673	3	0	3	8	24
AAU04658	3	0	3	8	24
AAU04642	3	0	3	8	24
APO40586	3	0	3	8	23
YP_003858591	3	0	3	8	24
QCC20721	3	1	3	-3	21
QGA70699	3	1	3	-3	20
AHY61344	3	2	2	-1	20
ASL68949	3	2	2	-1	20
YP_009361864	3	0	2	3	24
AIG13103	3	0	2	2	23
ASU90688	3	0	2	2	22
YP_009047211	3	0	2	2	22
YP_001039969	3	1	2	1	19
QHA24694	3	1	2	1	19
AWH65917	3	1	2	1	20
ANA96046	3	1	2	2	22
AIA62359	3	1	2	2	22
AWH65884	3	1	2	2	22
Q0Q4E6	3	1	2	2	22
YP_001039960	3	1	2	2	22
YP_173242	2	-1	2	5	12
YP_003029852	2	-1	2	6	17
YP_209238	2	-1	2	6	18
AAB86821	2	-1	2	6	17
NP_045302	2	-1	2	6	17
BAJ52884	2	0	2	5	17
YP_005454249	2	0	2	5	16
YP_009555245	2	0	2	5	16
AAY68302	2	0	2	5	16

ARC95207	2	0	2	5	16
BAF75637	2	0	2	5	16
ACX46856	2	0	2	5	16
P10527	2	0	2	5	16
ACJ66950	2	0	2	5	16
ABG89284	2	0	2	5	16
AHN64778	2	0	2	5	17
ACB30189	2	0	2	5	16
ABI94003	2	0	2	5	16
AZU96330	2	0	2	5	16
YP_009019186	3	-1	1	3	22
YP_009256201	3	-1	1	3	23
AFH58015	2	-2	1	5	18
AFG19745	2	-2	1	4	21
AMB66493	2	-2	1	5	22
YP_003771	1	-1	3	0	16
YP_009328939	1	-1	2	0	18
QHA24669	0	-2	1	-1	17
APD51503	0	-1	2	-1	16
NP_073556	0	-1	2	-2	16
YP_009194643	0	-1	2	-2	13
AOI28262	0	-1	2	-2	14
ATI09441	0	-1	2	-2	14
ASL24656	1	0	-1	4	23
AFE85966	1	-2	-2	2	20
YP_001351688	1	0	-1	4	20
QCX35174	0	0	-1	0	18
YP_009201734	0	-1	0	3	14
QHA24675	0	-1	-1	3	23
YP_001718609	0	-2	-1	3	17
QHA24714	0	0	-1	-1	19
YP_006908646	0	0	-1	-1	18
QCX35164	0	-1	1	0	16
AIA62275	0	0	0	-1	17
YP_009199794	0	0	0	-1	16

Supporting Table 2. NLS and NES motifs charges and nucleocapsid protein charge of coronavirus strains considered.

Name	Genomes (human viruses)	Genomes (all viruses)	Category
MERS-CoV	284	561	High-CFR
SARS-CoV	92	324	High-CFR
SARS-CoV-2	273	118	High-CFR
HCoV-HKU1	39	574	Low-CFR
HCoV-NL63	60	991	Low-CFR
HCoV-OC43	171	365	Low-CFR
HCoV-229E	25	61	Low-CFR
Total	944	2994	Human:<br>High-CFR: 649; Low-CFR - 295<br>All:<br>High-CFR: 1003; Low-CFR: 1991

Supporting Table 3. Number of CoV genomes considered in the analysis.

Supporting Figures

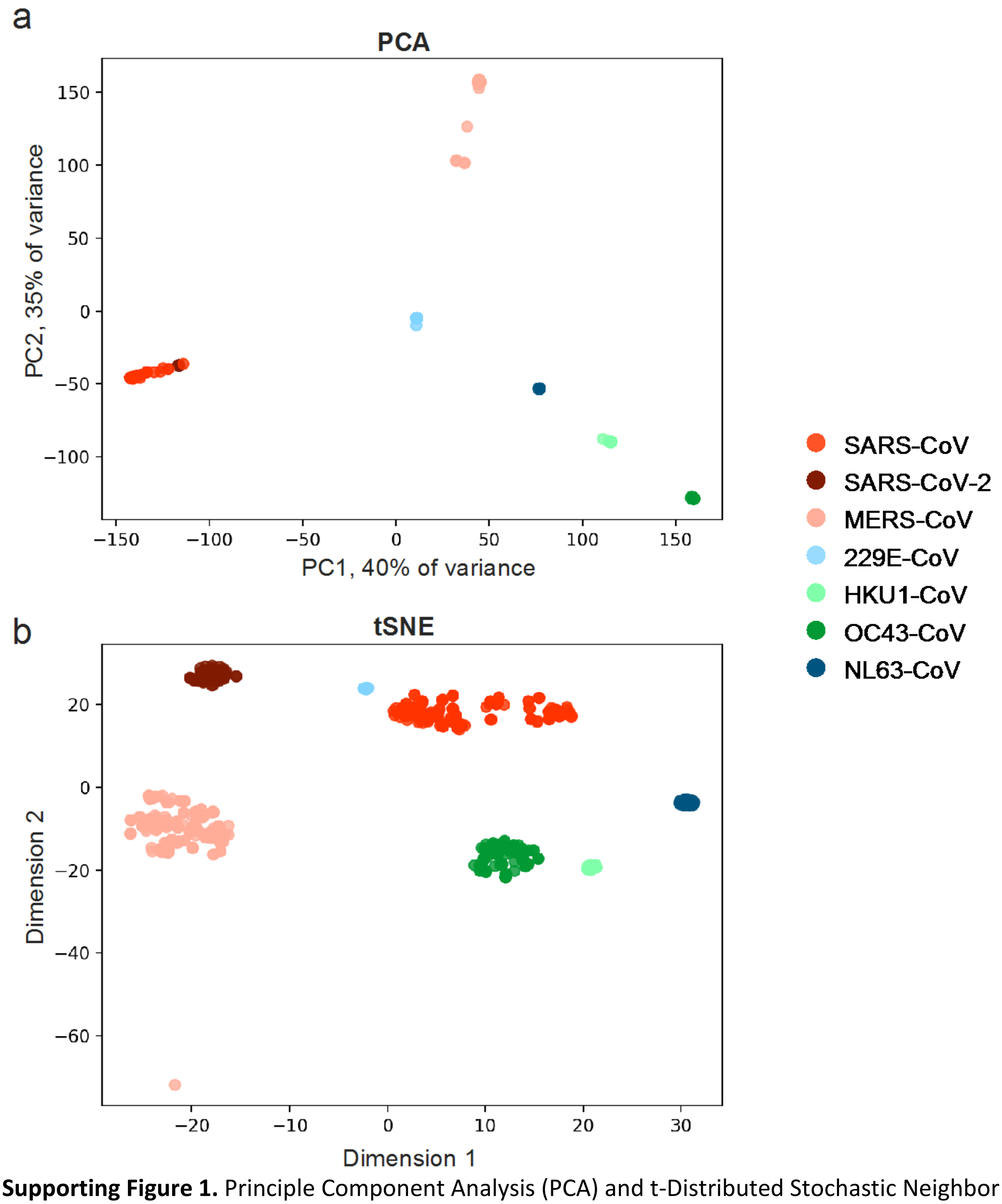

Supporting Figure 1. Principle Component Analysis (PCA) and t-Distributed Stochastic Neighbor Embedding (tSNE) of the encoded nucleotide alignments (where a gap is encoded with '1' and a nucleotide with '0').

In both panels, the red shades denote high-CFR CoV (all of which are betacoronaviruses), blue shades denote low-CFR alphacoronaviruses, green shades denote low-CFR betacoronaviruses. Both analyses show that the CFR trait has a stronger influence on genome clustering than phylogeny, and that there is more variability within high-CFR CoV in comparison to the low-CFR CoV. **(a)** PCA scatter plot, with PC1 (X-axis) vs. PC2 (Y-axis) obtained with PCA analysis of the encoded nucleotide alignments. The explained variance by each PC is noted in the axes. **(b)** tSNE scatter plot, with Dimension1 (X-axis) vs. Dimenstion2 (Y-axis) with 2-dimensions output.

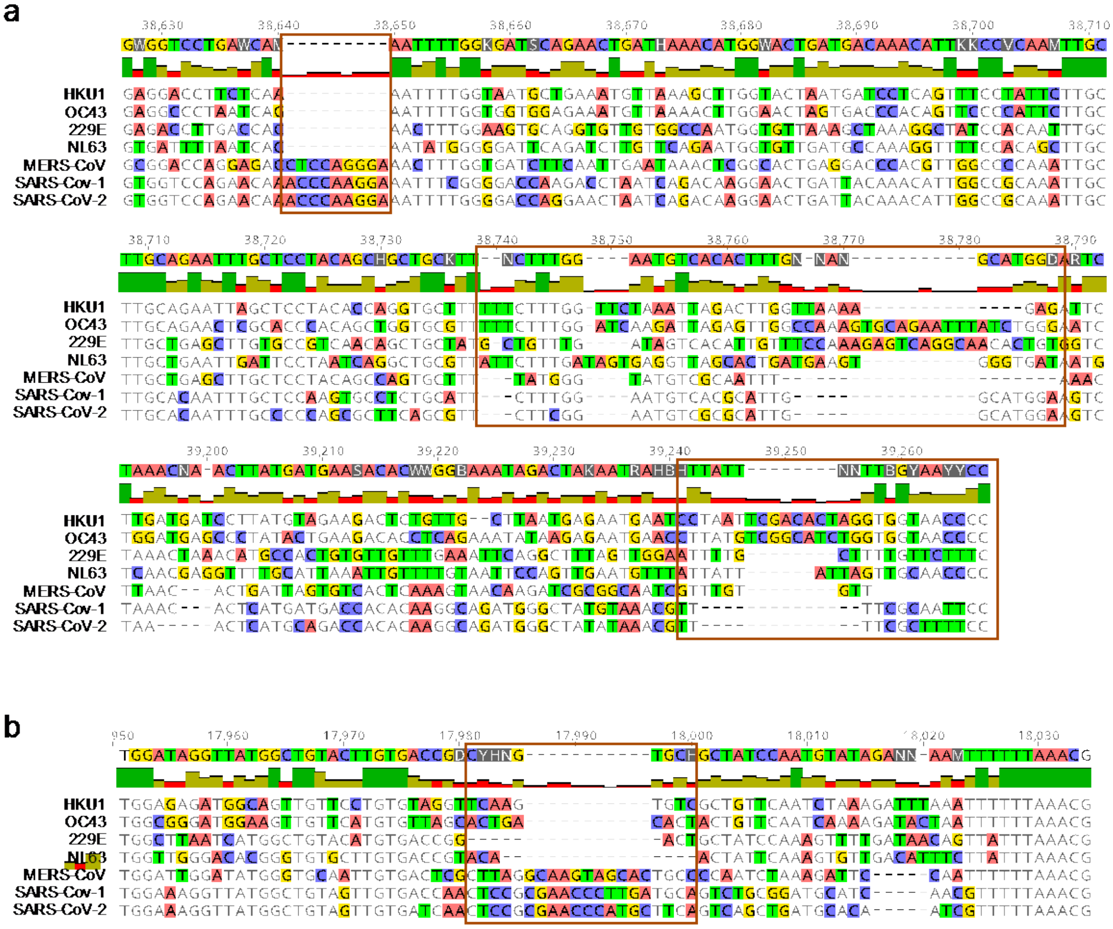

Supporting Figure 2. (a) The locations detected within the nucleocapsid protein from the nucleotide genome alignment. **(b)** The location detected within the spike protein from the nucleotide genome alignment.

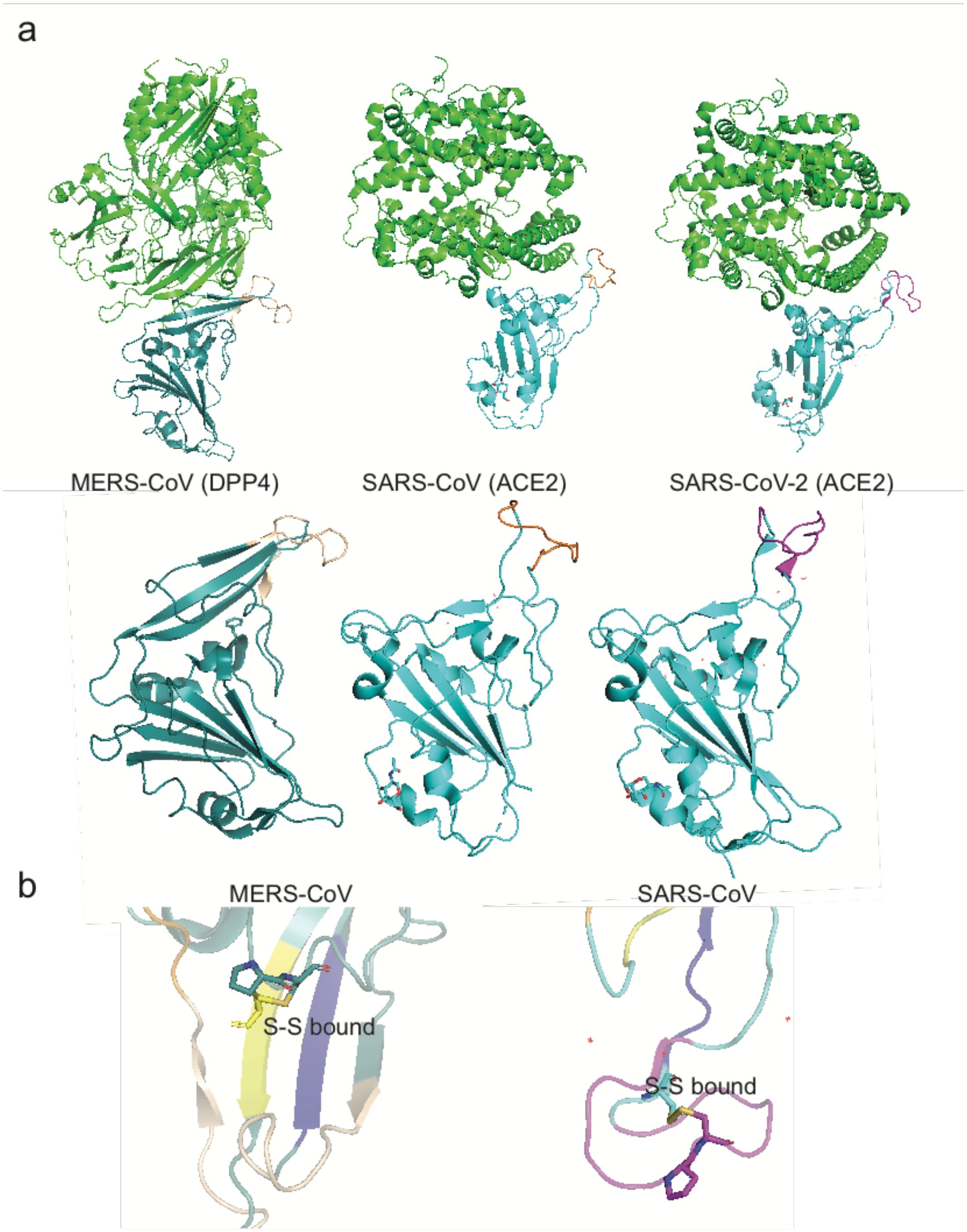

Supporting Figure 3. (a) Complete structures of the receptor-binding motifs of SARS-CoV, SARS-CoV-2 and MERS (blue) and the human receptor that they bind to (green) **(b)** Close-up of the Structures of the receptor-binding motifs of SARS-CoV and MERS-CoV. The inserts are highlighted, and the disulfide bonds are shown.
